# Classification Learning of Latent Bruise Damage to Apples Using Shortwave Infrared Hyperspectral Imaging

**DOI:** 10.3390/s21154990

**Published:** 2021-07-22

**Authors:** Jean Frederic Isingizwe Nturambirwe, Willem Jacobus Perold, Umezuruike Linus Opara

**Affiliations:** 1 Eresearch Office, DVC—Research and Innovation, University of the Western Cape, Private Bag X17, Bellville 7535, South Africa; fisingizwe@uwc.ac.za; 2 SARChI Postharvest Technology Research Laboratory, Faculty of AgrSciences, African Institute for Postharvest Technology, Stellenbosch University, Private Bag X1, Stellenbosch 7602, South Africa; 3 Department of Electrical and Electronic Engineering, Stellenbosch University, Private Bag X1, Stellenbosch 7602, South Africa; wjperold@sun.ac.za; 4 UNESCO International Centre for Biotechnology, Nsukka 410001, Nigeria

**Keywords:** machine learning, bruise detection, classification model, latent damage

## Abstract

Bruise damage is a very commonly occurring defect in apple fruit which facilitates disease occurrence and spread, leads to fruit deterioration and can greatly contribute to postharvest loss. The detection of bruises at their earliest stage of development can be advantageous for screening purposes. An experiment to induce soft bruises in Golden Delicious apples was conducted by applying impact energy at different levels, which allowed to investigate the detectability of bruises at their latent stage. The existence of bruises that were rather invisible to the naked eye and to a digital camera was proven by reconstruction of hyperspectral images of bruised apples, based on effective wavelengths and data dimensionality reduced hyperspectrograms. Machine learning classifiers, namely ensemble subspace discriminant (ESD), k-nearest neighbors (KNN), support vector machine (SVM) and linear discriminant analysis (LDA) were used to build models for detecting bruises at their latent stage, to study the influence of time after bruise occurrence on detection performance and to model quantitative aspects of bruises (severity), spanning from latent to visible bruises. Over all classifiers, detection models had a higher performance than quantitative ones. Given its highest speed in prediction and high classification performance, SVM was rated most recommendable for detection tasks. However, ESD models had the highest classification accuracy in quantitative (>85%) models and were found to be relatively better suited for such a multiple category classification problem than the rest.

## 1. Introduction

Bruise damage in fruit is very common and known to be one of the most contributing factors to degradation and loss of quality in fruit [1,2]. A wide range of preharvest and postharvest factors contributing to fruit bruising susceptibility and incidence have been reported [3,4,5]; however, bruise damage continues to occur due to inherent susceptibility of produce and inadequate application of control measures. The presence of bruising leads to reduced market acceptability of fresh produce and postharvest loss along horticultural value chain due to downgrading or outright rejection, thereby contributing to wastage and associated negative socio-economic and environmental impacts [6,7]. Extensive research on assessment [4,8,9] and non-destructive detection [10,11] of bruise damage has been conducted over the past few decades generating a certain level of understanding of the phenomenon and how to manage it [4]. Nevertheless, challenges remain, including that of effective and fast detection of such defects at early stages of development. Furthermore, bruised fruit undergo accelerated ripening and senescence, which necessitate the need for further detection and control measures [12].

The term ‘early detection’ of bruise damage in fruit material is related to the assessment carried out at early stages of bruise development. The term carries a sense of controversy in meaning, since the development is benchmarked based on visual assessment of the bruise whereby, in some background colors [1], bruises might not be noticeable and yet, be present, even externally. In many research reports, early bruise detection refers to an assessment conducted soon after (typically within a few hours) bruise induction [13,14].

For example, in a study on detecting ‘early’ bruises using thermal imaging combined with hyperspectral imaging, a cylindrical weight of 0.2 kg was used to induce bruise damage in peaches by dropping it from heights of 200 and 400 mm 1 h before assessing the bruised samples [15,16]. Considering such a drop as nearly free, the energy that was used to bruise the peaches would range from 0.39 to 0.78 J. While investigating the detection of fresh bruises (2 h old) in apples, using short wave infrared hyperspectral imaging, Keresztes et al. (2016) induced bruises by applying an impact energy of 0.41 J [17]. Elmasry et al. (2008) used a 250 g flat steel plate from a 10 cm drop height to induce bruises in McIntosh apples, which is estimated to be approximately 0.25 J [18]. Recently, Li et al. (2018) developed segmentation method to investigate the detection of early (1 h old) bruises induced by applying an impact energy of approximately 0.49 J, based on hyperspectral images. In another study of modelling apple bruise susceptibility under the influence of temperature, radius of curvature and acoustic stiffness, though the focus was not on early bruises, these were established at impact energies as low as 0.048 J and assessed after 48 h [19]. Since the manifestation of bruises depends on the type of fruit (attributes such as firmness, color, etc.), the energy applied to induce the damage and time elapsed thereafter, it would make sense to add to the definition of ‘early bruise’, bruises that have yet to manifest to visual inspection (latent bruises).

In all the above-mentioned cases where the non-destructive detection of early bruises in fruit was investigated, the bruising energy level was greater than 0.2 J and the definition of early bruises practically meant fresh bruises. However, it was found prior to this work, that bruises could be produced in apples, even at energy levels lower than commonly used in research experiments and damage could be embedded at a latent state. In such a case, however, traditional methods of assessing bruise volume and susceptibility, which are mainly based on visual aspects, would be highly challenged and likely unreliable.

On the bright side, there are many instruments that have shown potential for objectively detecting bruises, and near infrared (NIR) based techniques are at the forefront, for they offer more convenience and flexibility, which is desirable for industrial application. NIR based techniques are already commercially available for quality screening of foodstuff. Additionally, developments in spectral data-driven solutions are ongoing, which are likely to help achieve the requirements for effective detection of various latent defects, such as damage and pathological infection, among others [20,21].

The hyperspectral imaging (HSI) technique has been proven as versatile in detecting defects in fruit and plant material in general [22,23,24], for it offers both imaging and spectroscopic capabilities. Particularly, the uses of HSI for early detection of bruise damage has seen progress as per recent reports of application to peaches [14], mango [13] and apple [16,17,25], among others.

Therefore, an HSI system operating in the wavelength range of 900–2500 nm was used in this project with the aim to assess the feasibility of detecting latent bruise damage.

In this work, early detection of bruises in apple fruit was investigated by monitoring fruit samples exposed to impact forces, before and until bruise marks become evident to visual inspection. ‘Golden Delicious’ apples were used since they provide a high visibility of surface injuries and therefore allow for good assessment of bruise manifestation.

The objectives were to determine the state of bruise latency as it relates to impact energy and proven by objective image processing and visualization, to assess the detectability of such latent defects using classification learners of HSI data and evaluate the performance of derived binary and quantitative bruise classification models.

Impact bruise damages to apple were created at energies below the threshold found in the literature resulting in soft damage, undetectable by the naked eye. Visualization of bruises at specific wavelengths highlighted their subdermal existence and machine learning classification models were used to establish the feasibility of bruise segregation from healthy tissue to aid in rapid detection for sorting and grading.

## 2. Materials and Methods

### 2.1. Fruit Material and Bruising Experiment

Fruit material was sourced from a local retail market for locally grown fruits. In total, 36 Golden Delicious apples, free from visible defects, were selected from a set of packs and split into two batches for use in calibration (Batch 1) and validation (Batch 2), respectively. Three apples were used per bruising level for each batch, thereby resulting in six bruises per level per batch at a given scanned time during the entire experimental period.

Bruise damages were created on two opposite sides, in a middle area between the pedicel and peduncle on each fruit, by dropping a steel ball (63.79 g) onto the fruit in the equatorial area, after which bruised fruits were stored at room temperature (25 °C, 65% Rh) for nearly an hour prior to image acquisition. The impactor was dropped from different heights ranging from 0.02 to 0.32 m to create levels of bruise severity, L1 to L6, as denoted in Table 1. Assuming the fall was nearly free, impact energies applied on the fruit surface were calculated according to the method in [26] and ranged approximately from 0.013 J to 0.200 J (see Table 1), with respect to drop heights. It should be stressed that the three lowest energy levels used (L1–L3) were lower than the lowest experimental impact energy (0.048 J) previously found in the literature [19], by nearly a quarter. In this manner, boundaries of bruising energy were pushed in order to create latent bruise damage.

### 2.2. Hyperspectral Imaging System

An HSI unit from the Central Analytical Facility at Stellenbosch University was used for all the image acquisitions. The system is equipped with a line scanning hyperspectral camera (HySpex SWIR-384 from HySpex, Oslo, Norway) operating in the short-wave infrared (SWIR) range, from 930 to 2500 nm, in steps of approximately 5.45 nm, which results in 288 contiguous spectral wavebands in total. A moving sample stage allows for varying sample speed, an illumination unit consisting of two DC regulated light sources capable of delivering up to 150 W each and provide a stable (0.1% regulation, 0.1% noise) light intensity. The light sources are positioned at two opposite sides towards the sample, focusing the illumination to a line overlapping with the camera field of view. The system operation and image acquisition were carried out using ‘Breeze’ software (version 2019.1.0, Umeå, Prediktera, Sweden) installed on a computer running the Windows 10 operating system.

### 2.3. Image Acquisition

Prior to image acquisition the system was set up as follows. The distance between sample and camera was set to 20.5 cm; the grey standard was fixed at 68 mm from above the moving stage in alignment with the sample surface; the integration time was set to 3000 μs and the saturation of the grey standard set to 50%. The scanned length along the stage was 10 cm with a reference collection time of 60 s. Images of bruised apples were acquired on fruits moving at a speed of 5.47 mm/s using a ‘HySpex SDK’ camera lens with 30 cm focal length and a 95 mm field of view. The image acquisition was performed at 100 frames/s, with 404 frames per image and 384 pixels per line, setting the hypercubes at 404 × 384 × 288 in size. Both bruised sides of the fruit samples were scanned separately and used as individual samples. Additional to image acquisition within the first hour of bruising, images were also taken after 6, 18, 48 and 72 h to record the temporal bruise development in fruits initially bruised at low impact energy, as presented in Table 1 under the “Times scanned” column. Given that the main focus of was to assess the feasibility of detecting latent bruises, at higher impact energies such as L5 and L6, bruises were already severe enough withing a day after impact. Therefore, the latter were only scanned at fewer and earliest inspection times than at lower impact levels.

### 2.4. Image Analysis

Three dimensional hyperspectral images were imported into Evince software (version 2.7.10, Prediktera, Sweden) for pre-processing and background removal. The background was removed by interactively separating (selecting, excluding and reconstructing) the background pixels from the fruit pixels from a principal component analysis (PCA) based contour plots, applied on hypercubes. Segmented images were exported to a MATLAB (version R2019a, Mathworks, Natick, MA, USA) recognizable format for further processing; converted into MATLAB’s dataset objects (DSO) for subsequent use in unfolding 3D hypercubes into 2D data matrices, a technique of dimensionality reduction without loss of information [27]. The exported images were converted into hyperspectrograms for use in subsequent image reconstruction to visualize bruises at latent stage. Image reconstruction was done based on individual principal components (PC) generated to constitute hyperspectrograms, and using a MATLAB based conversion software tool (HyperspectrogramsGUI) available on request from the authors [28]. The hyperspectrograms method is useful in reducing dimensionality of hyperspectral data, making it easier to handle huge amounts of data, which is typical in hyperspectral imaging [22]. Additional to data visualization using image reconstruction from hyperspectrograms, MATLAB Image Processing Toolbox’s image player, was also used for visualization of wavelength specific images that best highlighted the well contrasted bruises. Furthermore, the regions of interest were extracted manually, which included 186 bruised areas and 287 areas of unbruised tissue. From all regions of interest corresponding average spectra were extracted for use in subsequent classification tasks.

### 2.5. Bruise Detection

In order to evaluate the implementation of discerning bruises at their latent stage, three aspects were established. First, bruise detection models were evaluated for their performance at predicting latent bruises. Classification models that encompass all used levels of severity were trained and tested on predicting the status of latent bruises. The models detect differences between bruised and sound tissue and are tested on latent bruises to decide whether they are recognized as bruises or not.

A second aspect that was investigated is the effect of temporal evolution of bruises on the detection model’s performance. The detection model, trained on all data, were tested on latent bruises at different scanned times.

Thirdly, a quantitative model that specifically considers bruise severity levels as categories was built in order to establish a framework for the identification of bruises quantitatively. Classification models were built to differentiate between these categories and were tested on samples at latent levels that were previously excluded from training.

**Figure 1:**
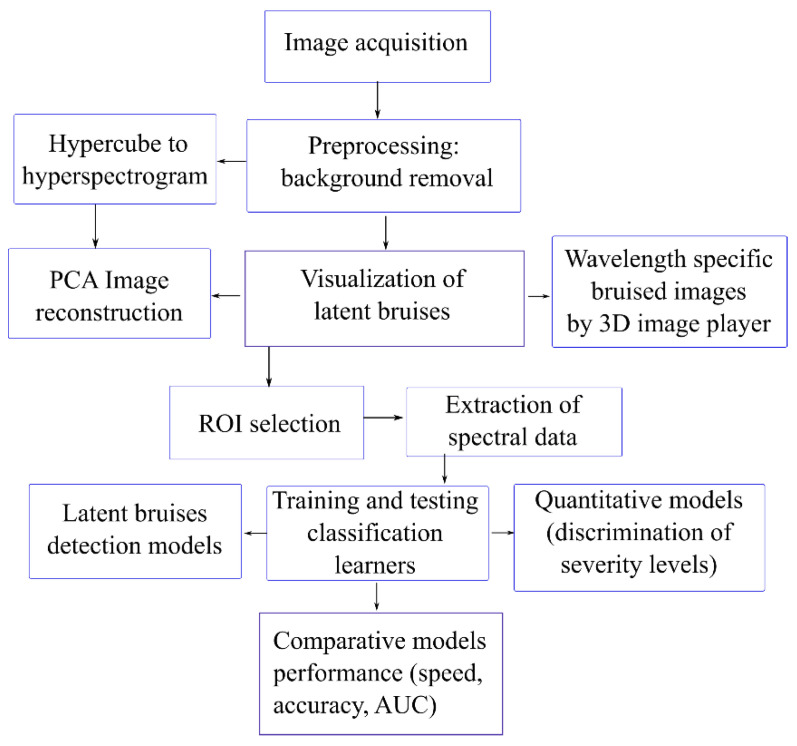
Graphical diagram for the analytical workflow followed in this study.

Classification models were built using learning algorithms available in the machine learning toolbox of MATLAB software, including decision trees, naïve Bayes, support vector machine, nearest neighbors and linear discriminant analysis and ensemble subspace discriminant algorithms. Only the best performing models were reported, some of which are described in the next section. In Figure 1, a schematic diagram of the analytical workflow that was followed is summarized.

### 2.6. Description of Classification Learners

The Support Vector Machine (SVM) algorithm for classification aims to maximize an optimal hyperplane as a decision function. The basic SVM deals with two-class situations, whereby the created hyperplane for separating data is defined by a number of margins to the nearest data points, also known as support vectors [29]. SVM has a reputation of high efficiency at avoiding issues of overfitting, which is typical of high-dimensionality data, such as that generated by HSI systems. It is known for excellent performance in classification and prediction [30]. In multiclass situations, some methods of combining multiple two-class SVMs are used. These methods include building all possible combinations of “one against one” two-categories classification problems and using either a voting strategy or an acyclic graph for deciding a sample’s correct class, or building all possible “one versus all the rest” dual-category problems in training and applying a decision function to determine a new sample’s category [31]. In this work SVM models were developed using a quadratic kernel function and a “one versus one” method.

In k-Nearest Neighbor (kNN), classification is performed by computing the Euclidean distance between a sample and each of the other samples in the training set. Once k nearest ones are found, the unknown is classified to the class that has most members among these neighbors [32,33]. A nonlinear classification algorithm, kNN is known to perform reasonably well in multiclass problems [34]. In this work preference was given to the “fine kNN” (FKNN) algorithm, using 1 neighbor and equal Euclidean distance weights.

The LDA method employs the Mahalanobis distance to estimate linear decision boundaries, which are defined in order to maximize the ratio of between-class to within-class dispersion [35], under the assumption that variance-covariance matrices of the classes are equal [36]. LDA is a popular method in chemometrics for its effectiveness of pattern recognition in multivariate data analysis.

Ensemble predictors combine results from many base learners, also referred to as ‘weak learners’, into one of higher performance, using methods such as bagging, subspace, boosting, etc. In Ensemble Subspace Discriminant (ESD), a random selection of features in the subspaces and a majority voting (between the predictors) rule are used to build the ensemble of learners and to adopt the classification result [37]. The ESD-based classification in this work used 30 learners and a 128-subspace dimension.

## 3. Results

### 3.1. Spectral Characteristics

**Figure 2:**
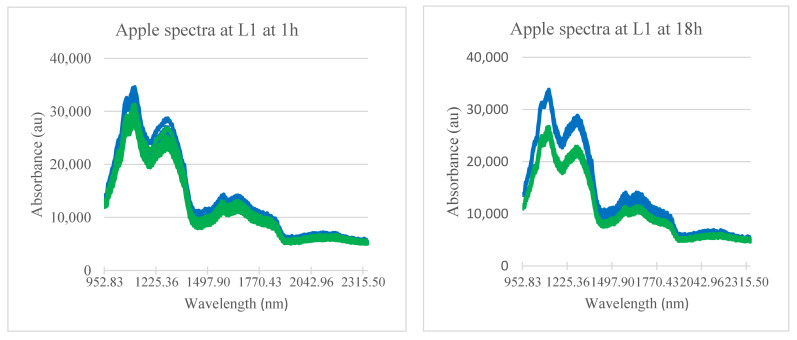

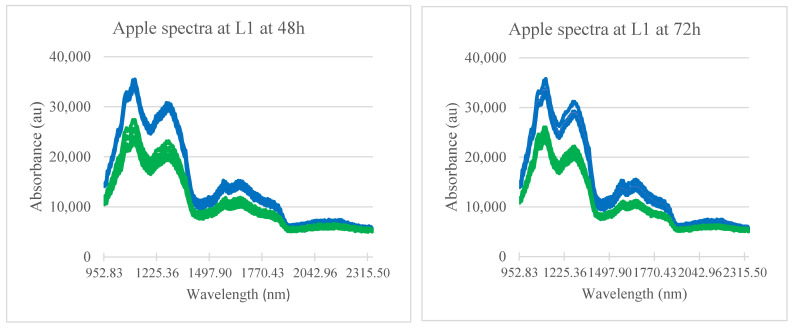
Temporal variation in spectral profiles of bruised (upper cluster: blue) and sound (lower cluster: green) apple tissue over 3 days. L1; lowest level of bruise severity used.

A representation of spectral characteristics of bruised and sound apple tissue is presented in Figure 2. Each spectrum is an average of spectra corresponding to pixels within a selected bruised or non-bruised area or region of interest (ROI).

Spectra showed higher levels of absorbance in bruised tissue than that of sound ones, around the peaks at 1130 and 1285 nm for latent bruises that are less than 1 h old. With time, the spectral bands from 952 to 1377 nm and 1550 to 1850 nm showed an increasing gap between spectra of bruised and sound tissues. This increasing gap can be attributed to changes in chemical composition in bruised areas, which are also associated to tissue deterioration subsequent to bruising injury [38]. These wavebands appearing to be related to bruise development are in agreement with the wavelength ranges (1000–1300 nm) reported as appropriate for detecting bruises in ‘Golden Delicious’ apples [39] and they contain the wavelengths (1131.0 nm and 1376.5 nm) previously selected as effective in the detection of bruises to various apple cultivars [40].

### 3.2. Spatial Characteristics

In order to show that bruises could be detected at their latent stage, a data visualization was carried out by reconstruction of hyperspectral images in a manner that highlights the bruises. Two methods were used for this purpose, namely hyperspectrograms based image reconstruction [27] and using single wavelength images captured using the Image player tool from MATLAB software.

**Figure 3:**
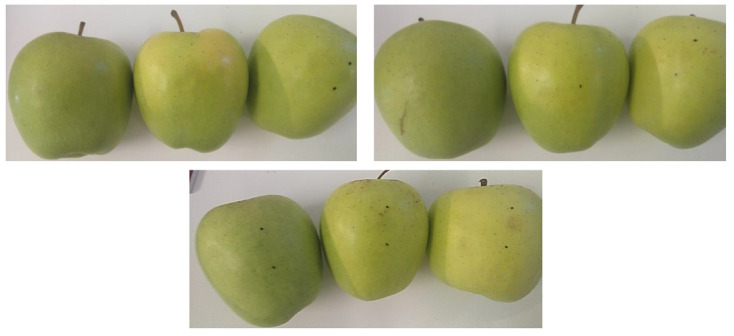
RGB images of bruised ‘Golden Delicious’ apples; bruises are mostly invisible to an eye and digital camera.

A representation of RGB images (size: 4208 × 2368 pixels) of apples bruised at L1 and L2 after 1 h of bruising were taken by a smartphone camera (model ‘Redmi Note 2′, 13 MP, f/2.2, PDAF), using a MediaTek camera application, in JPG format, is shown in Figure 3. Bruises were practically invisible to the naked eye and indistinguishable from sound tissue as per the RGB images acquired by the digital camera and shown in Figure 3. However, a slight tissue discoloration at the bruised location could be noticed on some, but not all apples at L2, when inspected with high attention from 1 h after impact.

To prove whether latent bruises were successfully encoded in the hyperspectral images, two methods were used for visualization of possible latent defects. First, upon removal of background pixels using PCA, images were exported from Evince software to a MATLAB compatible format of hypercubes and used in the ‘video viewer app’ of MATLAB’s image processing toolbox to select wavelengths that yielded the best contrasted bruises.

**Figure 4:**
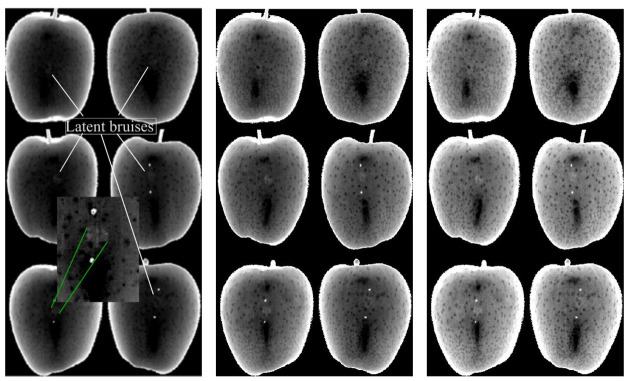
Images of apple samples at 1203 nm (**left)**, 1340 nm (**middle**) and 1667 nm (**right**) showing bruises at L1.

**Figure 5:**
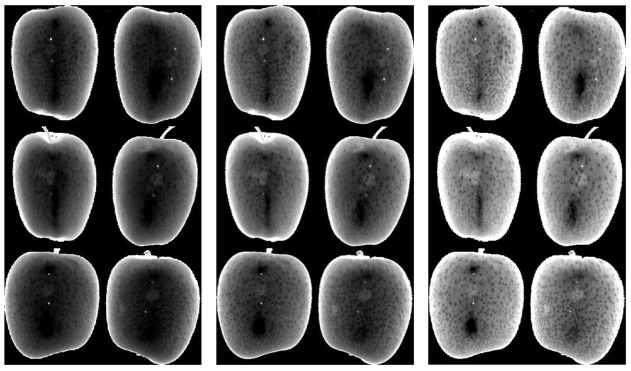
Images of apple samples at 1203 nm (**left**), 1340 nm (**middle**) and 1667 nm (**right**) showing bruises at L2.

Figure 4 and Figure 5 show the visualization of bruises at wavelengths which best highlight latent bruises, for L1 and L2, respectively. Wavebands around 1203, 1340 and 1667 nm provided the most resolved bruises without any need for postprocessing for image enhancements.

It is noticeable from Figure 4 and Figure 5 that the bruises (brighter circular marks in the middle of the fruit images) were more encoded at L2 than at L1, which is to be expected, as the higher the bruising impact, the more distinguishable bruises will be. Even though the bruises at latent states, such as L1 and L2, seem visually insignificant and harmless, they develop and become visibly significant over time, as shown in studies that considered longer periods of time (6 days) than used in this study [41].

Bruises at 1340 nm were relatively the most distinguishable from the rest of the apple surface. Images at 1203 nm had the least contrasted bruises, but still distinguishable by exploration of pixel intensity values. In the study of bruise detection by Kersesztes (2016), in the SWIR range, two spectral regions (water absorption peaks around 1400 and 1700 nm) were selected as most effective wavelength regions in distinguishing bruises from sound tissue of apples [17]. These values overlap with and validate the selected wavebands in the current study.

**Figure 6:**
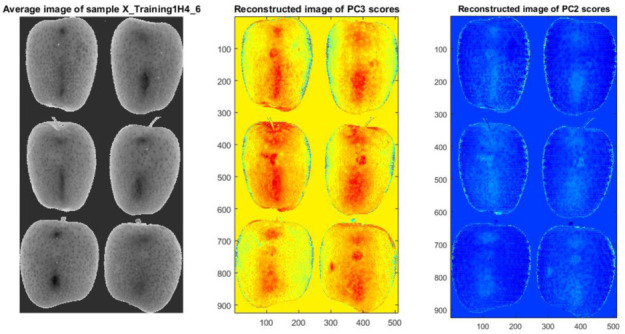
Image reconstruction from hyperspectrograms showing bruises at L2; average image (**left**), PC3 image (**middle**) and PC2 image (**right**).

Secondly, the hypercubes were converted to “Dataset object” (DSO) format and loaded in the hyperspectrogram conversion software [28] to generate the hyperspectrograms based on features generated by applying PCA. To be specific, a hyperspectrogram was created by joining the frequency distributions curves of the scores, Q residual, Hotelling T^2^ and the loading vectors, in sequence. By limiting the principal components (PCs) to 3, this data dimensionality reduction method reduced each hypercube of dimension *H* = (404 × 384 × 288) to a one-dimensional signal of size *h* = (3 × 288) + (404 × 384 × 5), which captures both the spectral and spatial information in the original images. Therefore, the compression ratio of data from a hypercube to the corresponding hyperspectrogram was *H/h* = 57.5. Image reconstruction was selectively performed based on the constitutive frequency distributions that make up the hyperspectrograms. Images with optimal features of defects were retained, as shown in Figure 6. Bruises at L2 were visibly contrasted from sound tissue in PC2 (right) and PC3 (middle) scores images, whereas on the average image (left) there is no indication of bruised area. The clear visibility of the reconstructed bruises suggests that the hyperspectrograms method, which has previously been used successfully in detecting bruises to apple during their temporal development [41], could also be a fit method to detect latent bruises in apples.

**Figure 7:**
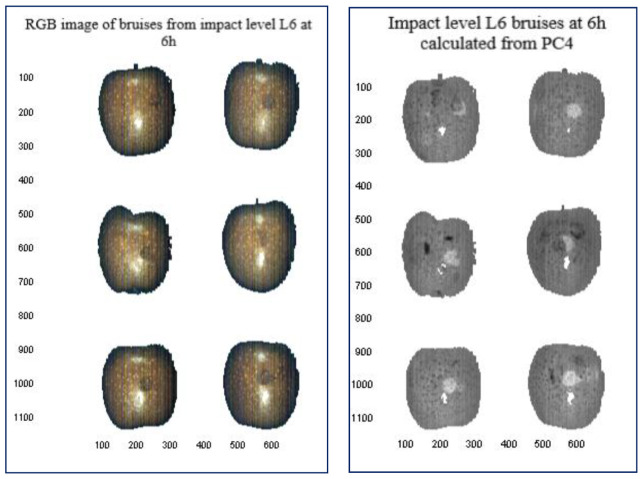
Image reconstruction showing bruises at L6 after 6 h; RGB color (**left**) and calculated bruises PC4 images (**right**). Bruises appear as dark grey spots on the RGB images and as light grey marks on the PC4 images.

While bruises were not visible to the naked eye, at high impact levels both the Hyperspectral RGB image and the PC based image show highly contrasted bruises (light grey marks) against the surrounding sound apple tissue. In Figure 7, PC images (right) are shown alongside hypercubes average RGB image (left) for severe bruises at L6. The gray scale image was generated using PCA mapping and is based on the fourth principal component (PC4).

### 3.3. Latent Bruise Detection Models

Classification models were built using various classification learners available in the machine learning toolbox of MATLAB software and exported to MATLAB workspace to be subsequently used in predicting external test data. The training set consisted of all data acquired over five scanned times on all six levels of bruise severity (186 spectra), including sound tissue (287 spectra) extracted from regions of interest (473 observables in total). Half the latent bruise data subset for a severity level that is targeted for prediction (i.e., either L1 or L2) was excluded from training and used as test set for testing models on new unknown data. The global model was trained on all samples available and tested on half the 1 h old samples at L1–L3 combined with the same fraction from healthy samples. In order to avoid model overfitting, 10-fold cross-validation was used in the training step. Classifier’s performance was evaluated with respect to accuracy and area under receiver operating characteristic curve (AUC). The best performing four were retained and reported in Table 2.

The results in Table 2 show that quadratic-kernel support vector machine (Q-SVM) had the best performance in all regards, followed by fine k-Nearest Neighbour (F-KNN) and Linear Discriminant Analysis (LDA). Prediction of the state of bruises in L1 (latent bruises induced at lowest energy level) was relatively lower than that of bruises in L2 (latent bruise induced with second lowest energy). This should be expected, as the higher the severity of the bruise, the easier it is to detect. Overall, latent bruises were predicted with high accuracy, ranging from 89.6% to 100% and a generally perfect value for AUC, which suggests high performance for all classifiers. It was noticed that ensemble classifiers were slowest to train and yielded slightly different results for each run, therefore an average of a model accuracy was calculated over five runs.

### 3.4. Influence of Temporal Evolution on Detection of Latent Bruises

Fruits with latent bruises were monitored over a period of 3 days of bruise development and during this time, they were scanned at 5 temporal instances (1, 6, 18, 48 and 72 h). To investigate the effect of time after bruise induction on the detection accuracy of latent bruises (L1 and L2), classification models to distinguish bruised from sound tissue were built using various classification learners. For each temporal instance in consideration (Table 3), 50% of the corresponding samples were excluded from training for use later in testing the model, while the remaining 50% was included in training. Training was carried out on all non-excluded L1 and L2 samples scanned from 1 h all the way to 72 h, and model performance was evaluated based on their accuracy, as summarized in Table 3.

Overall, the models yielded high prediction accuracy in both training and testing with an apparent increase in model accuracy on tested samples with increasing age of bruises, for F-KNN and ESD. A slight misclassification was obtained for 1 h old samples. However, all misclassified samples were originally from the non-bruised category (false positives) and thus, all defects were correctly classified, which is the most desirable outcome in practice. With increasing time, the misclassification rate reduced overall, and at 48 h the tested sample accuracy was 100%. However, Q-SVM models were not influenced by the time elapsed whatsoever. The accuracy in training and testing remained the same, regardless of the age of defects, and was highest in training. A comparison of classifiers based on prediction speed (the number of observations processed per second during validation of the model) showed that the Q-SVM classifier was consistently the fastest, whereas ESD was the slowest. Prediction speed values normalized against the highest value from all classifiers are shown in Table 3. The demonstrated superior performance of the SVM makes it recommendable for detection of latent bruises in apples. This comes as no surprise, SVM algorithms have previously been reported to have the highest preference for the analysis of hyperspectral data [31].

### 3.5. Quantitative Prediction of Latent Bruises

Quantitative (severity) prediction of bruise damage is important because it provides a categorization of fruit with respect to bruise severity, which is an important aspect for the fruits grading task. With such information one would be able to determine the appropriate use of produce; such as packing fruits that are free from defects for the local market, long distance transport or export; mildly bruised fruits for processing and severely bruised fruits for animal feed. An attempt to predict bruises (including latent ones) based on their quantitative aspects was carried out, whereby classification models were built by considering bruise severity (six levels) as the dependent variable (Y) that embodies the quantitative feature. The multiclass calibration models were developed based on a batch of samples representing six levels of bruising assessed at approximately 1, 6, 18, 48, and 72 h after bruise induction.

Class labelling was completed by combining bruising level and time after bruise induction, whereby the notation “Lxyz” stands for bruising level “Lx”, where 1 ≤ x ≤ 6 recorded after “y” or “yz” hours. Training the models was carried out with 10-fold cross validation on all classes and tested at 1, 6 and 18 h separately, on half the samples at low-level classes (L1 to L3), which was excluded from training. As a result, ensemble subspace discriminant (ESD) yielded the best prediction performance overall, followed by linear discriminant analysis (LDA) and lastly, fine k nearest neighbors (F-KNN) (see Table 4), whereas the speed of prediction and training was highest for F-KNN. The latter had the highest misclassification rate with 1 to 6 h old bruises, whereas for bruises at 18 h it achieved a categorization with as high an accuracy as achieved with ESD.

Even though the inclusion of time evolution implies that the differences between the so formulated bruise class levels are subtle, the classifiers achieved high accuracy as indicated in Table 4. In a real application scenario, a few bruise severities such as latent, visible and severe, for example, may be sufficient depending on the goals for grading. Such few categories summarizing a large range of severity levels would preserve the classifier’s performance and ensure successful distinction between classes with high accuracies.

**Figure 8:**
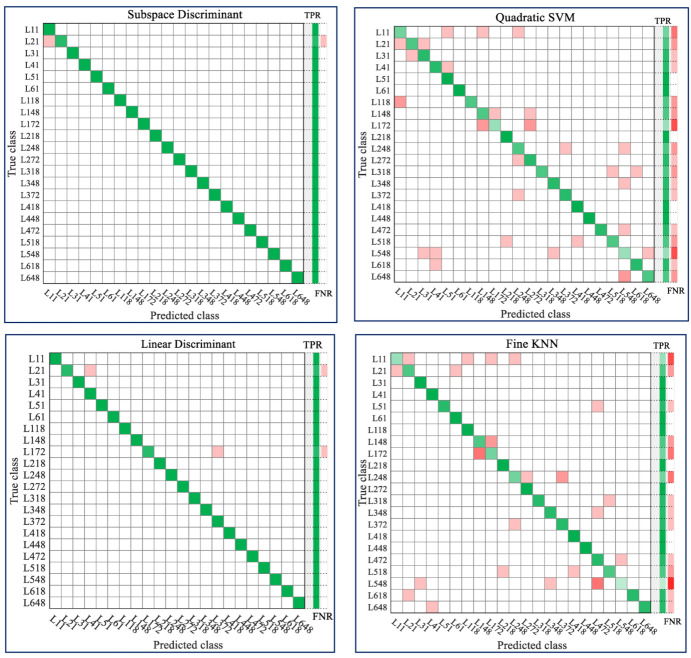
Confusion matrix to showcase classification performance for bruise severity by ESD (**top left**), QSVM (**top right**) LDA (**bottom left**) and FKNN (**bottom right**).

In Figure 8 the confusion matrices of classification models for quantitative aspects of bruises are shown for three classifiers, namely ESD, QSVM, LDA and F-KNN. The true classes are the categories initially assigned to the respective samples, whereas the predicted classes represent the categories that the samples are found to belong to as a result of the classification task. The model development included six levels of bruise severity evolving over time at instances including 1, 18, 48 and 72 h and a 10-fold cross-validation method was used to avoid model overfitting. The highest true positives to total positives ratio (TPR) and lowest false negatives to total positives ratio (FNR) were obtained by the ESD classifier, followed by LDA. True positives are depicted using a green color, while false negatives are marked in red with darker hues corresponding to higher rates. From the confusion matrices, it is noticed that in most cases of misclassification the predicted class was closely similar to the true class, which suggests that in cases where classes were agglomerated into larger groups resulting in fewer categories, the misclassification would also be reduced.

## 4. Discussion

Successful application of approaches that used spectral average of ROIs were reported in previous studies of bruise detection [1,40]. The models developed in this work are based on spectral data, provided that the bruised region to have already been located on the fruit. This work extended such application particularly to the detection of latent bruises as well as exploiting the possibility of implementing a quantitative prediction model for bruise damage. In an industrial application scenario, it would be convenient to detect bruised regions using methods such as watershed segmentation [14], other thresholding methods [40] or pixel-based bruise segregation [17,42], prior to evaluating the severity of the defects as demonstrated in this work. However, these bruise segregation methods are yet to be tested on latent bruises. It is also worth mentioning that in methods for locating bruised regions that do not take the spectral dimension of hypercubes into account, such a task would be highly challenging, or at least computationally costly, thus a quick determination of optimal wavelengths for visualizing latent bruises, similar to this work, would be very useful. Alternatively, methods of image synthesis [43] can be useful in highlighting faint features such as latent bruises and enhance the efficiency of search algorithms.

The analytical workflow used in this work combined various software applications. However, in real life applications it would be advantageous to work with a single end to end software platform, which would combine search and segregation of regions of interest (e.g., region with defect), extract features (e.g., spectral data) and predict the state and/or severity of the bruises. Such end-to-end solutions are unknown to the authors and likely non-existent currently. Future work should put an effort into developing such solutions. The use of deep learning methods [44] is one such approach that could enable compact workflows for detecting defects on whole images of fruit requiring fewer to no steps of prior selection of ROIs than in this study, and it is intended to explore this in future work. It is important to note that deep learning methods require extensive computational infrastructure and much larger datasets of images than was used in this work.

Results showed that the behavior of classifiers in two-class problems for bruise detection was different to that in multi-class problems for quantitative models. F-KNN was the fastest in quantitative models, whereas SVM was the fastest in detection tasks. Though SVM achieved the best performance in terms of accuracy and prediction speed for detection tasks, it did not perform well for quantitative models. However, it was observed that when the number of response classes (31 groups) was reduced to nearly half (14 groups), the performance of SVM models was improved, which suggests that the lower the number of categories in a classification task, the higher SVM was likely to perform. The results suggest that SVM is recommendable for latent bruise detection tasks while for quantitative models, ESD and LDA would be a better fit to achieve high accuracy. However, a loss in prediction speed was involved.

This lower prediction speed, especially for ESD, is only relative to the other tested algorithms. When translated to applications where such speed is crucial, such as inline sorting, the requirements by the latter can still be met by the algorithms. For example, in a typical fruit sorting system, speeds from 5 samples/s [17] up to 10 samples/s (about 1 to 2 m/s) in commercial sorting lines [45] are used, while in-field defect detection has been proven feasible at a speed of 0.5 m/s [46]. Within such speed requirements one has to account for all the analytical steps of a detection task, such as data (image) acquisition, processing, feature extraction, ROI selection and classification. The speeds reported here (>400 samples/s) are only related to and sufficient for the classification part. The analytical steps prior to classification have been shown to be achievable using some versions of scanning systems and researchers [46,47] have made confident reports about the current technical advancements to help achieve such and more requirements for the agricultural and food industry.

Bruise severity modelling is an important prerequisite for tasks of grading fruit based on level of damage. However, it is not commonly reported on in postharvest research. The modelling approach presented here can be adopted for the detection of various other defects, especially for severity estimation models. Although this work was based on an imaging system, the application of this methodology has extensive relevance to spectroscopic systems as well.

In this work, a single background (bright green) was considered, but future work should expand the training dataset to include other aspects, such as various backgrounds and catching angles for light during scanning. The findings show that latent bruises can be accurately detected using hyperspectral imaging data after quick search and determination of regions of interest (bruised area).

One important aspect of machine learning models that is required for successful application tasks is the ability of the models to generalize. To ensure this is implemented, calibration models were tested on new, unseen samples, initially set aside from the overall dataset; however, there is room for improvement, and it is suggested that future work should consider data augmentation techniques such as in [48,49] to further improve on the generalization ability of the models such as these developed in this study.

## 5. Conclusions

Bruise damages were established on Golden Delicious apples, which have a bright green epidermis allowing for clear visibility of external defects, such as bruises. The existence of latent bruises on apples was proved by comparing RGB camera images, firstly to images reconstructed based on hyperspectrograms, which are reduced data embedding features from hypercubes, and secondly, to fixed wavelengths images with optimally contrasted latent bruise marks. While RGB images showed no visible bruises, image reconstruction made the bruises evident. Detection of latent bruises was carried out using various machine learning classifiers and resulted in high detection accuracy with quadratic-kernel support vector machine leading in performance and prediction speed. Quantitative aspects of bruises were modelled by considering bruise level of impact and time after bruising as factors contributing to variations in bruise severity. The ensemble subspace discriminant classifier was recommended as best of the tested algorithms for quantitative models, based on its higher rate for correct recognition of new (test) samples. However, higher classification accuracy was associated with bruise detection models, both for global models and latent bruises, than in quantitative classification models. The findings in this work prove that quantitative aspects in bruise detection models are implementable. The work presented here should serve as a benchmark for the industry in implementing a quantitative assessment of bruise damage and latent damage detection. Future work will focus and on improving the model generalization ability by implementing data augmentation methods to new data and on applying cutting edge classification methods such as deep learning algorithms to the current problem, to constitute a more compact pipeline for image processing and classification learning.

## Figures and Tables

**Table 1:** Parameters of the bruising experiment.

Bruising Parameters			Number of Individual Bruises				Total Number of Bruise Samples
Drop Height (m)	Bruise Level Notation	Energy of Impact (J)	Batch 1	Times Scanned	Batch 2	Times Scanned	
0.020	L1	0.013	6	4	6	2	36
0.040	L2	0.025	6	4	6	2	36
0.07	L3	0.044	6	4	6	2	36
0.126	L4	0.079	6	4	6	1	30
0.219	L5	0.137	6	3	6	1	24
0.319	L6	0.2	6	3	6	1	24
	TOTAL						186

**Table 2:** Performance of detection model for latent bruises in ‘Golden Delicious’ apple.

	Training Accuracy (%)	AUC	Test Accuracy (%)	Prediction Speed (Observations/s)	Classification Learner
Detect L1	100	1	94.4	4200	Q-SVM
	99.1	0.99	89.9	2200	F-KNN
	99.4	1	89.9	2200	LDA
	99.6	1	100	400	ESD
Detect L2	100	1.00	100	2700	Q-SVM
	98.4	0.98	94.4	1500	F-KNN
	98.8	1.00	100	3600	LDA
	99.6	1	100	400	ESD
Overall model	100	1.00	100	1900	Q-SVM
	98.7	0.98	96.2	1600	F-KNN
	99.6	1.00	96.2	1800	LDA
	99.6	1.00	100	440	ESD

**Table 3:** Temporal influence on detection of latent bruises.

Model	Training Accuracy (%)				Test Accuracy (%)				Prediction Speed (Normalized) *			
	1 h	6 h	18 h	48 h	1 h	6 h	18 h	48 h	1 h	6 h	18 h	48 h
LDA	98.7	94.8	99.4	95.5	94.4	100	88.9	100	0.78	0.56	1	0.50
F-KNN	98.7	98.7	95.5	96.1	88.9	94.4	100	100	0.61	0.42	0.52	0.68
Q-SVM	99.4	99.4	99.4	99.4	100	100	100	100	1	1	1	1
ESD	100	97.4	98.7	98.7	94.4	100	100	100	0.16	0.16	0.11	0.16

*: Prediction speed is expressed in “observations/s”, ratios normalized to the highest value per column are shown.

**Table 4:** Quantitative prediction of latent bruises.

Bruise Level	Classification Accuracy		Classifier
	Training	Test	
L11–L31	69.8	66.67	F-KNN
	93.7	88.89	ESD
	89.3	77.78	LDA
L16–L36	69.5	44.4	F-KNN
	92.1	88.89	ESD
	90.4	66.67	LDA
L118–L318	66.9	88.89	F-KNN
	94.4	77.78	ESD
	87.6	77.78	LDA
Global L1–L6	75.8	48.15	F-KNN
	98.5	62.96	ESD
	97.0	74.07	LDA

## Data Availability

The data presented in this study are openly available in Kikapu, a Figshare repository at doi:10.25379/uwc.14906691.
